# Evaluating the performance of gene-based tests of genetic association when testing for association between methylation and change in triglyceride levels at GAW20

**DOI:** 10.1186/s12919-018-0124-y

**Published:** 2018-09-17

**Authors:** Jason Vander Woude, Jordan Huisman, Lucas Vander Berg, Jenna Veenstra, Abbey Bos, Anya Kalsbeek, Karissa Koster, Nathan Ryder, Nathan L. Tintle

**Affiliations:** 1 Department of Mathematics and Statistics, Dordt College, 498 4th Ave. NE, Sioux Center, IA 51250 USA; 2 Department of Computer Science, Dordt College, 498 4th Ave. NE, Sioux Center, IA 51250 USA; 3 Department of Biology, Dordt College, 498 4th Ave. NE, Sioux Center, IA 51250 USA

## Abstract

Although methylation data continues to rise in popularity, much is still unknown about how to best analyze methylation data in genome-wide analysis contexts. Given continuing interest in gene-based tests for next-generation sequencing data, we evaluated the performance of novel gene-based test statistics on simulated data from GAW20. Our analysis suggests that most of the gene-based tests are detecting real signals and maintaining the Type I error rate. The minimum *p* value and threshold-based tests performed well compared to single-marker tests in many cases, especially when the number of variants was relatively large with few true causal variants in the set.

## Background

Methylation data continues to grow in popularity owing to both its increasing availability (decline in cost) and biological relevance, a result of increasing hypotheses about the contribution of epigenetic effects to the genetic architecture of common human diseases. This rapid rise in popularity has meant that there are few “best practices” for the analysis of genome-wide epigenetic data. However, many of the current analytic approaches for methylation data are informed by the more mature field of genome-wide association studies (GWAS).

For many years, the use of multimarker tests of genetic association has been a popular alternative to single-marker tests in GWAS. Multimarker tests have the potential ability to aggregate weaker individual signals across a biologically related set of markers, reduce the substantial multiple testing penalties required for GWAS, and directly connect statistical testing with functional biological units (eg, genes or other meaningful sets). The rise in the popularity of next-generation sequencing data and the subsequent ability to easily and inexpensively measure rare genetic variants has made multimarker tests a necessity by requiring the aggregation of signals from rare variants in order to improve statistical power to a reasonable level.

Prior work by our group [1, 2], and many others [3, 4], evaluated numerous strategies for summarizing marker-level genetic association statistics across biologically informed sets. For example, burden tests are well known to lose power when testing sets of markers containing both risk-increasing and risk-decreasing variants, whereas variance components tests are robust to these situations and mixtures of both methods can sometimes yield “optimal” power [2, 3]. We have identified a test statistic that is particularly robust to situations where the majority of markers in the set are noncausal, which can be near optimal when combined with a variance components test [1].

In this paper, we evaluate the application of novel gene-based tests of association when analyzing simulated genome-wide methylation data as compared to single-marker tests. We choose test statistics and evaluate their behavior in light of recent methodological results on gene-based tests for rare genetic variants (see previous paragraph). We evaluate the performance of novel gene-based tests across different simulated data sets provided as part of GAW20, and as compared to direct application of “standard” single-marker testing approaches.

## Methods

### Sample population and variables

We analyzed the simulated data set provided as part of GAW20 and were aware of the “answers” (simulation parameters) when conducting this analysis. The sample consisted of 670 individuals for whom all analyzed variables were available. We considered 7 covariates (age; observation center; smoking status; International Diabetes Federation [IDF] mass spectrometry DX client [MSDX]score; fasting time at baseline; high-density lipoprotein [HDL] at baseline; and triglyceride level at baseline). The primary response variable of interest was change in triglyceride (TG) level from baseline (visit 1 or 2) to follow-up (visit 3 or 4). For variables with up to 2 measurements at baseline or follow-up (HDL [baseline], TG [baseline, follow-up]) we used the average value if both measurements were available, or the only available measurement if only one was available.

### Models

We used a 2-stage modeling process. The first stage resulted in 200 models (one for each of the 200 simulations provided). The second stage resulted in 654,755 models (one for each single-nucleotide polymorphism [SNP] that passed standard GWAS quality control [QC] criteria: Hardy-Weinberg Equilibrium *p* value > 1 × 10^− 6^, minor allele frequency > 1%, SNP missing data rate < 5%).

The *lmekin* function from the *coxme* package in R [5] was used to predict the change in log-transformed TG levels (*y* = ln(*followup*) − ln(*baseline*)). In cases where two separate TG measurements were available for either follow-up or baseline, we natural-log (ln)-transformed the data before averaging. Change in ln-transformed TG levels was predicted by the 7 covariates listed earlier, baseline ln-transformed TG levels, and the familial relationships in the model (which were accounted for through the use of the kinship matrix). For each of the 200 simulations, we then saved the resulting “residual” value (${r}_i={\widehat{y}}_i-{y}_i$) for each of the *i* = 1,…,670 individuals in our analysis.

The second stage predicted the residuals (${r}_i^{\prime }s$from stage 1 based on the number of minor alleles (*SNP*_*j*_ = 0, 1, 2) and methylation scores (*CPG*_*j*_ ∈ [0, 1]) along with an interaction term between *SNP*_*j*_ and *CPG*_*j*_, with a separate model for each *SNP*_*j*_, *CPG*_*j*_ pair. In particular, the second stage model for the *SNP*_*j*_, *CPG*_*j*_ pair was:

1 $$ r={\beta}_{S_j}{SNP}_j+{\beta}_{C_j}{CPG}_j+{\beta}_{SC_j}{SNP}_j{CPG}_j $$

*SNP*_*j*_, *CPG*_*j*_ pairs were made by pairing each SNP passing QC to its nearest cytosine-phosphate-guanine (CpG) site resulting in 654,755 pairs, with some CpG sites assigned to multiple SNPs. The only exception to this pairing strategy was for 3 SNPs with major effects (see next paragraph for details) which were assigned to the “causal” CpG site, which was not necessarily the nearest CpG (in all cases these were within 12,500 bp). We note that the model in eq. (1) is informed by the true simulated data model for the data provided as part of GAW20, in which SNP effects are moderated by methylation of nearby CPG sites.

### Gene selection

Our analyses focused on 3 distinct subsets of genes. First, the GAW simulated data set includes 5 genes (hereafter, *major effect genes*) containing (or within 50,000 bp of) a causal SNP with heritabilities of 0.025, 0.05, 0.075, 0.10, and 0.125. Second, the GAW simulated data set contains 34 genes containing exactly 1 causal SNP with heritability of 0.001 (hereafter, *minor effect genes*). Third, we randomly selected 39 other genes from the remaining list of 16,604 genes not containing causal variants (hereafter, *noncausal genes*). Thus, a total of 78 genes were considered in our analyses.

Sets of SNPs were assembled for each gene, *k* = 1,…,78. In particular, for most genes, all SNPs contained within the start–stop positions of the gene (based on human genome build 18 [hg18]) were considered “part of” the gene. The exceptions to this were 3 major-effect SNPs that were not located within a gene. In 2 cases, the causal SNP was within 50 kb of the nearest gene and so was added to the set of SNPs within the gene (*SIPA1L2* and *MSRB2*). In the final case, where the nearest major-effect SNP was not within 50 kb of the nearest gene, we created a synthetic gene that included the SNPs within 50 kb of the SNP (*SYNTH1*).

### Gene sets

We also considered 5 sets of variants that were not solely defined by gene boundaries. One of these sets (CAUSAL5) consists of only the 5 causal variants with heritabilities of 0.025 or larger (*major effect genes*) (to act as a positive control). Two sets, UNION5 and UNION2, are, respectively, the union of all 5 causal genes and the union of *LYRM4* and *HS3ST3A1*, and thus contain 5 and 2 causal variants, respectively. NOISE5 and NOISE2 also have 5 and 2 causal variants, respectively, but the rest of the variants are either noncausal or minor causal.

### Gene-based test statistics

We evaluated 6 structurally different gene-based test statistics in addition to a “standard” single-marker test. For each gene, *k*, a new statistic, *G*, was created by using various methods of combining the *p* value from the F-statistic test on the overall model significance of eq. (1), over all *m* SNP-CpG sites assigned to the gene. Thus, *m* distinct *p*_j_ values were combined into a single value (*G*_*j*_). Table 1 shows the 6 methods we used to compute *G* as a function of *p*.

**Table 1:** Overview of gene-based test statistics considered

	SNP*CPG, *G*_*SC*_
Sum of natural log-transformed *p* value (Sum (ln *p*))	${\sum}_{j=1}^m\ln \left({p}_j\right)$
Sum of negative squared natural log-transformed *p* value (Sum (−(ln*p*)^2^))	${\sum}_{j=1}^m-{\left(\ln {p}_j\right)}^2$
Minimum *p* (Min *p*)	$\underset{j\in \left\{1,\dots, m\right\}}{\mathit{\min}}\left({p}_j\right)$
*p* value threshold 0.01 (*p*T 0.01)	$$ {\sum}_{j=1}^m\left\{\begin{array}{cc}-{\left(\ln \left(\frac{p_j}{0.01}\right)\right)}^2& \mathrm{if}{p}_j\le 0.01\\ {}0& \mathrm{if}{p}_j>0.01\end{array}\right. $$
*p* value threshold 0.05 (*p*T 0.05)	$$ {\sum}_{j=1}^m\left\{\begin{array}{cc}-{\left(\ln \left(\frac{p_j}{0.05}\right)\right)}^2& \mathrm{if}{p}_j\le 0.05\\ {}0& \mathrm{if}{p}_j>0.05\end{array}\right. $$
*p* value threshold 0.10 (*p*T 0.10)	$$ {\sum}_{j=1}^m\left\{\begin{array}{cc}-{\left(\ln \left(\frac{p_j}{0.1}\right)\right)}^2& \mathrm{if}{p}_j\le 0.1\\ {}0& \mathrm{if}{p}_j>0.1\end{array}\right. $$

Choices of *G* were informed by prior research (see *Background* for details). In brief, the *sum of ln p* is informed by Fisher’s method for combining tests and burden tests (although robust to different effect direction), *sum of squared ln p* is informed by variance components tests, and *min p* is informed by recent research on test statistics highly robust to large proportions of nonassociated statistics. We proposed 3 threshold-based tests that attempt to put a threshold on the “noise” of noncausal SNPs through a *p* value threshold of either 0.01, 0.05, or 0.10. We used negative ln-transformations of *p* in line with prior research (eg, Fisher’s combined probability test). The benefit of the threshold approach is that any *p*_*j*_ above the threshold value will have no effect on the summation across the *m* SNP-CpG sites. Thus all SNP-CpG sites that would be considered not statistically significant on their own at the threshold level will contribute nothing to *G*, while other SNP-CpG sites will contribute according to the square of the natural log of their scaled *p* value.

### Permutations

Permutations were used to assess the statistical significance of *G*. Briefly, the residual values from stage 1 were computed separately for each individual in each simulation. These residual values were permuted and then the permuted residual values were used to generate permuted *β* values in stage 2. We did 1000 permutations for each simulation considered, making sure to reuse the same shuffles for each SNP-CpG pair to preserve correlation structure between and across CpG sites and SNPs within each gene. Empirical *p* values were computed as the proportion of permuted values of *G,* which were more extreme than the observed value of *G*. We used a significance level of 0.05 for all tests, except single-marker tests which used a significance level of $\frac{0.05}{m_k}$ where *m*_*k*_ represents the number of SNPs, *m* in gene (or set) *k*, representing a candidate gene significance level.

## Results

### Performance across 200 simulations

In Table 2, performance for each gene-based test statistic, *G*_*SC*_, is provided, stratified by whether a gene (or set) contained 1 or more major causal variant, minor causal variants, or no causal variants. Performance is assessed by computing the proportion of genes with *p* values less than 0.05 across all genes and simulations, except for single-marker *p* values, which were evaluated using a Bonferroni-corrected significance threshold of $\frac{0.05}{m_k}$ where *m*_*k*_ represents the number of SNPs, *m* in gene (or set) *k*. For single-marker tests, genes containing 1 or more SNPs with a *p* value below the threshold were deemed significant. Table 2 illustrates reasonable control of the false-positive error rate as all methods detected less than 5% of genes containing no causal variants as significant. Genes containing minor causal variants were only detected slightly more frequently than genes containing no causal variants, and so we focus the remainder of our analysis on genes containing major causal variants.

**Table 2:** Proportion of times test statistic, *G*_*SC*_, was rejected (*p* < 0.05) across 200 simulations, by choice of test statistic and by type of gene

Statistic	Contains major causal variants	Contains minor causal variants	Contains no causal variants
Sum ln *p*	0.367	0.07	0.04
Sum −(ln*p*)^2^	0.398	0.06	0.04
Min *p*	0.431	0.03	0.02
*p*T 0.10	0.460	0.04	0.03
*p*T 0.05	0.469	0.04	0.03
*p*T 0.01	0.467	0.03	0.02
Single marker^a^	0.403	0.03	0.02

^a^Single marker test used a Bonferroni-corrected significance threshold of $\frac{0.05}{m_k}$

Tables 3 and 4 highlight the power of each SNP-CpG statistic, *G*_*SC*_, across the 5 major effect genes (Table 3) and synthetically created sets of SNP-CpG pairs (Table 4).

**Table 3:** erformance across major-effect genes

Gene	SNP heritability	No. SNP-CpG pairs	MAF of causal variant	Sum ln*p*	Sum −ln^2^*p*	Min*p*	*p*T 0.10	*p*T 0.05	*p*T 0.01	Single marker
SIPA1L2^a^	.125	141	0.11	0.35	0.42	0.73	0.58	0.65	0.72	0.69
SYNTH1^b^	.100	23	0.19	0.79	0.74	0.48	0.65	0.61	0.52	0.41
LYRM4	.075	63	0.10	0.17	0.18	0.22	0.23	0.22	0.25	0.21
HS3ST3A1	.050	29	0.41	0.24	0.22	0.21	0.24	0.24	0.21	0.19
MSRB2^a^	.025	32	0.14	0.72	0.72	0.41	0.65	0.59	0.48	0.39

^a^Nearest gene within 50,000 bp of major-effect SNP

^b^Artificial “gene” containing all SNPs within 50,000 bp of major effect SNP

**Table 4:** Performance across sets of SNP-CpG variant pairs containing major-effect variants

Gene	Total SNP heritability	No. SNP-CpG pairs	Sum ln*p*	Sum-ln^2^*p*	Min*p*	*p*T 0.10	*p*T 0.05	*p*T 0.01	Single marker
CAUSAL5	0.375	5	1.00	1.00	1.00	1.00	1.00	1.00	1.00
UNION5	0.375	288	0.73	0.86	0.74	0.93	0.92	0.89	0.70
UNION2	0.125	92	0.23	0.26	0.26	0.25	0.26	0.27	0.23
NOISE5	0.375	288	0.06	0.11	0.64	0.50	0.61	0.70	0.64
NOISE2	0.125	92	0.03	0.09	0.20	0.14	0.15	0.18	0.20

Table 3 demonstrates that for genes containing only a single, highly heritable variant single-marker methods perform reasonably well compared to gene-based methods. In 3 of the 5 cases (*SIPA1L2*, *LYRM4*, and *HS3ST3A1*), one or more of the threshold-based approaches (*pT*) and *min p* methods outperformed or performed similarly to single-marker methods, but averaging methods (*sum of ln p* and *sum of squared ln p*) performed comparably (*HS3ST3A1* and *LYRM4*) or worse (*SIPA1L2*). In 2 cases (*SYNTH1* and *MSRB2*), averaging methods outperformed the other methods, with threshold methods performing next best followed by *min p*, and single-marker methods performing worst. The *pT 0.01* and *min p* methods outperformed single-marker methods in all 5 cases.

As seen in Table 4, all methods performed well on a set containing only causal variants with high heritability (*CAUSAL5*), but once noncausal variants were added, the aggregating methods outperformed single-marker method (*UNION5*, *NOISE5*). A similar pattern was observed with sets containing 2 causal variants (*UNION2* and *NOISE2*).

## Discussion and conclusions

To date, few papers have considered multimarker (gene-based) approaches for methylation data. Our proposed approach to the aggregation of statistical evidence of phenotypic association across multiple SNP-CpG pairs serves as a proof-of-concept of this approach in candidate gene analyses investigating the moderating effects of methylation. In particular, in a candidate gene, versus genome-wide, context significance levels are higher and in line with those used here (0.05). Our analysis demonstrates reasonable false-positive rates, and generally good performance of multimarker methods on sets containing SNP-CpG sites with reasonably large effects. As is often the case in practice, the ability to detect markers with low heritability remains challenging.

In general, the patterns seen for the performance of multimarker tests of SNP-CpG pairs follow those for SNP-variant-based analysis methods. In particular, sets with lower numbers of variants and only a single causal variant were challenging for multimarker methods to detect, although averaging methods tended to outperform threshold-based and the *min p* methods. As the number of variants increased, threshold-based and the *min p* methods tended to outperform averaging type multimarker tests. As the number of causal variants in the set increased, multimarker tests performed better than single-marker tests. The threshold-based testing approaches are a reasonably novel approach to multimarker testing, and performed reasonably well as a robust intermediary to the *min p* method (optimized for large numbers of variants when few are causal) and averaging methods (*sum of ln p* and *sum of squared ln p*) (optimized for lower numbers of variants with multiple causal variants).

The GAW20 simulated data set only contained 200 simulations, and so our analysis was limited in the ability to draw broad conclusions about power and Type I error. Further work is needed to explore the widespread control of Type I error and power of multimarker tests for methylation data in more wide-ranging simulated data sets and in a genome-wide testing situation (lower significance levels). We also note that our choice to use a linear model containing an interaction term between methylation (CpG) and SNP was informed by the simulation model used in GAW20. While serving as a proof-of-concept for the multimarker analysis of methylation data, in practice, the test statistic used should be informed by the hypothesized biological mechanism of the effect of methylation. The model used here is a reasonable, although not necessary, hypothesis of this effect. Further work is needed to investigate other models and the performance of multimarker methods in those settings. Our results suggest the use of gene-based tests when investigating methylation-SNP impact on phenotypes; however, further testing is needed in more wide-ranging and comprehensive simulation settings.
